# Stress Wave Propagation in Two-dimensional Buckyball Lattice

**DOI:** 10.1038/srep37692

**Published:** 2016-11-28

**Authors:** Jun Xu, Bowen Zheng

**Affiliations:** 1 Department of Automotive Engineering, School of Transportation Science and Engineering, Beihang University, Beijing, 100191, China; 2 Advanced Vehicle Research Center, Beihang University, Beijing, 100191, China

## Abstract

Orderly arrayed granular crystals exhibit extraordinary capability to tune stress wave propagation. Granular system of higher dimension renders many more stress wave patterns, showing its great potential for physical and engineering applications. At nanoscale, one-dimensionally arranged buckyball (C_60_) system has shown the ability to support solitary wave. In this paper, stress wave behaviors of two-dimensional buckyball (C_60_) lattice are investigated based on square close packing and hexagonal close packing. We show that the square close packed system supports highly directional Nesterenko solitary waves along initially excited chains and hexagonal close packed system tends to distribute the impulse and dissipates impact exponentially. Results of numerical calculations based on a two-dimensional nonlinear spring model are in a good agreement with the results of molecular dynamics simulations. This work enhances the understanding of wave properties and allows manipulations of nanoscale lattice and novel design of shock mitigation and nanoscale energy harvesting devices.

Granular materials, due to their intrinsic discreteness, randomness and interaction diversity, exhibit a wide range of distinguished physical phenomena in mechanics, electromagnetism as well as quantum mechanics from macroscale to nanoscale^1^,^2^,^3^,^4^,^5^,^6^,^7^. The concept of force chain or force network acts as a powerful tool to visualize force (or stress, strain) distribution in solid granular media and bridges granular materials to complex network analysis with the aid of experimental methods such as photoelasticity and computer simulations, inspiring the novel design of stress wave controlling and energy absorption system^8^,^9^,^10^,^11^,^12^,^13^,^14^,^15^,^16^,^17^. Once orderly arranged, granular materials are able to exhibit unique wave behaviors and extraordinary (sometimes even counterintuitive) ability to tune stress wave thanks to tunable contact nonlinearity^18^. One-dimensional (1D) homogeneous chain of spherical granules, as the simplest arrangement, has been theoretically, numerically and experimentally proved to be capable of supporting the propagation of strongly nonlinear translational solitary wave^19^,^20^,^21^,^22^,^23^ governed by highly nonlinear Hertzian contact^24^. A solitary wave is a nonlinear dispersionless wave without any temporal evolution in shape and its phase speed is amplitude-dependent. Mathematically, weakly nonlinear solitary waves are the solutions of a class of nonlinear equations such as the Korteweg-de Vries (KdV) equation, sine-Gordon equation as well as the nonlinear Schrödinger equation^23^,^25^ and strongly nonlinear Nesterenko solitary wave is a solution of highly nonlinear wave equation first introduced for Hertzian chain by Nesterenko in ref. 19 and for general interaction law in ref. 22. In addition, desired wave behaviors can be obtained by purposely manipulating physical properties of the components of the above-mentioned strongly nonlinear system such as material, shape and size of grains. It makes granular materials a promising candidate for shock disintegration, energy harvesting, nondestructive testing, etc^26^,^27^,^28^,^29^,^30^,^31^,^32^,^33^,^34^,^35^,^36^,^37^,^38^,^39^,^40^,^41^,^42^. Wave properties of coherent tightly packed granular systems in 2D and 3D have also been investigated, for example, 2D square packing, curved channel, Y-shaped packing and ordered granular network^43^,^44^,^45^,^46^,^47^,^48^,^49^,^50^,^51^,^52^,^53^,^54^,^55^,^56^,^57^,^58^,^59^. Recently, inspired by these macroscopic studies, we have carried out investigations of the counterpart system at nanoscale, and were able to show that 1D arrayed buckyball (C_60_) system, resembling macroscale spheres, also supports strongly nonlinear Nesterenko solitary wave^60^. However, this solitary wave is both qualitatively and quantitatively different from that of macroscale system. On one hand, waves in nanoscale system is very sensitive to ambient temperature, which directly determines the level of thermal vibration that is harmful to nanoscale solitary wave propagation. On the other hand, governed by van der Waals interactions instead of the Hertz law in contact mechanics, the *force-overlap distance* relation of adjacent interacting nanoscale granules exhibits no linear term with a stronger nonlinearity than Hertzian law^61^, thus leading to a different *amplitude-wave speed* relation of the strongly nonlinear waves. We have established a semi-empirical nonlinear spring (NS) model to accurately describe nanoscale solitary waves at low temperature (10 K), as a simplification of intermolecular interaction as well as an analogue to Nesterenko solitary wave. Substituting buckyballs with carbon nanotubes (resembling cylindrical or tubal particles), we have demonstrated that 1D single-walled carbon nanotube (SWNT) system serves as a highly effective and reusable energy absorber^62^. Further, we studied a C_60_-SWNT hybrid system, having achieved a quantitative tuning of nanoscale solitary waves with good precision^63^.

In this study, we investigate stress wave behaviors in 2D buckyball system via molecular dynamics (MD) simulation, as an extension of our previous work on 1D nanoscale lattice. As coherent macroscale studies suggest, 2D constructions render more types of wave patterns, suggesting a very promising potential for stress wave-related applications. The presentation of our work is structured as follows. In Sec. 2, we first introduce the setup of investigated system, including the square close packing (*scp*) and the hexagonal close packing (*hcp*) system. In Sec. 3, we put forward a theoretical model to describe 2D problem. In Sec. 4, we present the results of MD simulations and numerical calculations based on the 2D NS model of the two packing modes. Finally, in Sec. 5, we present some concluding remarks and possibilities for future investigations.

## System descriptions

**Figure 1:**
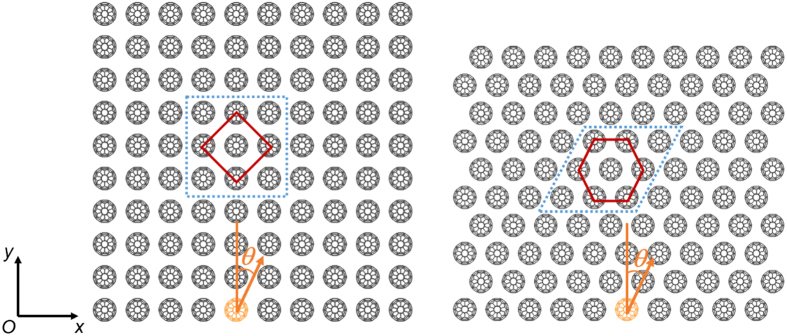
Schematics of 2D *scp* and *hcp* C_60_ system. The impactor molecule and information of impacting velocity are highlighted in orange.

In this study, we investigate two basic 2D uniform packing modes, i.e. *scp* and *hcp*, whose coordination numbers^64^ (or contact numbers) are 4 and 6 respectively and *hcp* is the densest packing mode possible (with a packing density of ~0.9609), as is illustrated in Fig. 1. In this paper, the size of investigated systems is chosen as 10-order. The shortest distance between two C_60_ molecules equals to the equilibrium spacing *d*_0_ = 10.05Ǻ^65^, corresponding to the close packing modes. A C_60_ molecule on one edge away from the corners is selected as an impactor to generate a stress wave with an initial velocity of 2000 m/s, a moderate impacting velocity for studying nanoscale impact dynamics. The impacting direction is determined by an impacting angle *θ*, defined as the angle between the velocity and the *y* axis (see Fig. 1). The MD investigation of stress wave behaviors of the system is carried out based on the open-source program LAMMPS (Large-scale Atomic/Molecular Massively Parallel Simulator)^66^ and visualized using molecular visualization program VMD (Visual Molecular Dynamics)^67^. To minimize the disturbance of thermal vibration, the initial temperature is set as *T* *=* 10 K and the systems are run for equilibrium in the canonical ensemble (NVT) for 3 ps, which is a sufficient time duration in this study (see Figure S1). Major investigations of wave behaviors are in the micro-canonical ensemble (NVE). More detailed simulation descriptions are provided in the Supplementary Information.

## Theoretical modeling

The dynamics of the 2D system is modeled by a 2D NS model, as a dimensional extension of 1D NS model. In this study, the values of both stiffness parameter *k* and nonlinearity index *n* are identical to the 1D NS model, whose theoretical background is briefly reviewed in the Supplementary Information. The Hamiltonian of the whole C_60_ system can be given as follows


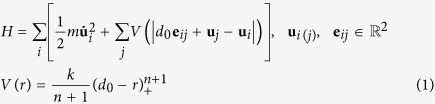


where *V*(*r*) is the potential function of two interacting C_60_ molecule with respect to distance *r*; **u**_*i*_ is the displacement vector from the initial position of *i*^th^ C_60_ molecule; **e**_*ij*_ is the unit vector pointing from *i*^th^ C_60_ molecule to *j*^th^ C_60_ molecule.

In numerical calculations of *scp* and *hcp* arrangements all interactions of a C_60_ molecule with 8 surrounding molecules are taken into consideration (see the regions surrounded by dashed lines in Fig. 1) without any symmetrical or collision mode-related simplifications. It is advantageous over previous numerical methods with respect to calculation precision^46^,^47^,^48^,^49^,^55^. The 2D equation of motion of *i*^th^ C_60_ can be given as


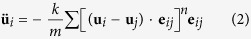


The numerical calculations are performed using a forth order Runge-Kutta algorithm to integrate the nonlinear ordinary differential equations^68^. As an initial condition, the displacement vector **u**_*i*_ of each C_60_ equals to zero vector and the velocity vector **u**_*i*_ is also zero vector except for the impactor, whose initial velocity is nonzero. The numerical calculation lasts 3000 fs each time.

## Results

We choose two impacting angles to study the impact responses of nanoscale lattice, i.e. *θ* = 0° and *θ* = 30°. The magnitude of particle velocity is extracted to characterize stress wave propagation. The investigation of wave behaviors in *scp* and *hcp* configurations is organized as follows: (a) particle velocity magnitude *v*_M_ distributions at given instants; (b) particle velocity amplitude *v*_Amp_ distributions; (c) comparison between MD simulation results and numerical calculation results, i.e. solutions of Eq. (2); (d) Discussions on the unique wave properties in *scp* and *hcp* configurations.

### Square close packing

**Figure 2:**
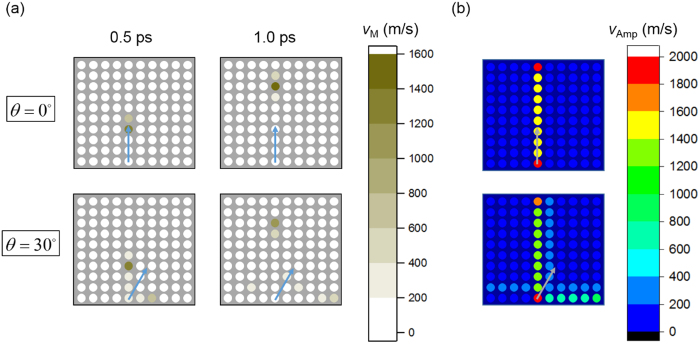
Stress wave distributions in *scp* system for different impacting angles. (**a**) Particle velocity magnitude distributions at given instants, i.e. 0.5 ps and 1.0 ps. (**b**) Particle velocity amplitude distributions.

Particle velocity magnitude distributions at given instants and particle velocity amplitude distributions of *scp* configuration under two different impacting angles are presented in Fig. 2.

As is illustrated in Fig. 2, stress wave in *scp* system is highly directional where the major portion of wave energy transmits through initially excited chains. In fact, waves traveling in initially excited chains are solitary waves, whose shape and wave speed remain constant as they travel (see Figure S2). Later we will show that these solitary waves are in good agreement with Nesterenko’s well-known 1D solitary wave in many aspects. Because of the highly directionality of *scp* packing modes and the dispersionless characteristics of translational solitary waves, there is potential for *scp* granular configuration to be applied to stress wave guidance and energy focusing etc.

**Figure 3:**
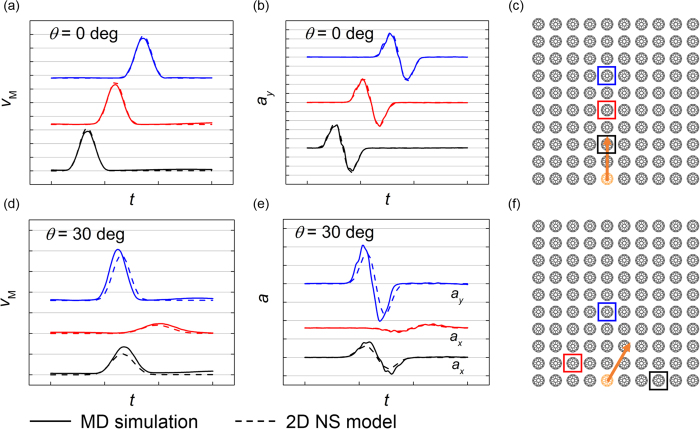
Comparison of particle velocity magnitude *v*_M_ and acceleration *a*_*x(y)*_ between MD simulations and numerical calculations in *scp* granular configuration for different impacting angles. *v*_M_ and *a*_x(y)_ of the C_60_s in squares are extracted and the colors of the curves and the squares are corresponding. The vertical scale is 5 × 10^15^ m/s^2^ and the horizontal scale is 500 fs. (**a**), (**b**) and (**c**) *θ* = 0°. (**d**), (**e**) and (**f**) *θ* = 30°. The results of MD simulations and numerical calculations based on the 2D NS model are plotted in solid lines and dashed lines respectively.

The comparison between MD simulations and numerical calculations of *scp* system is shown in Fig. 3, where the time histories of particle velocity magnitude and acceleration on certain direction of 3 representative C_60_s are compared.

According to the comparison in Fig. 3, the results of MD simulations and numerical calculations are in excellent agreement. To confirm that the prediction accuracy of NS model is not sensitive to the position of impactor C_60_ molecule, another comparison is made where the impactor is chosen as the C_60_ molecule at the bottom left, as is shown in Figure S3.

**Figure 4:**
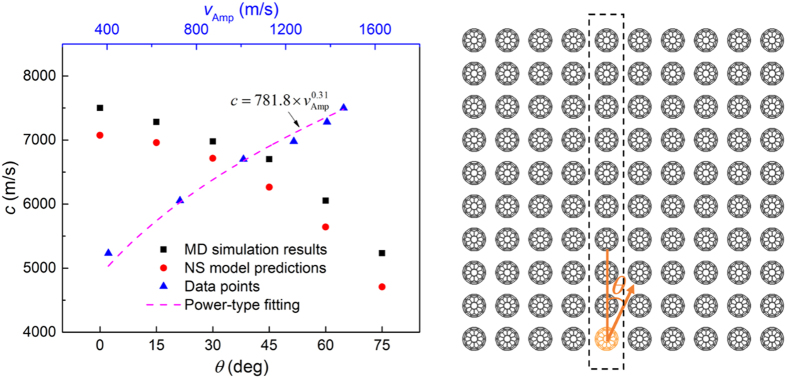
Wave speed of initially excited solitary wave as a function of impacting angle (force amplitude) or particle velocity magnitude.

A further exploration to the properties of excited solitary waves in 2D *scp* system is as follows. A set of MD simulations are conducted where the impacting angles range from 0 deg to 75 deg, resulting in a series of solitary waves of various force amplitudes (presented in Table S1) propagating along the C_60_ chain in the dashed square in Fig. 4. With the theory of 1D NS model (see the Supplementary Information), wave speed can by predicted by inputting wave amplitude and the predicted values match well with simulation results (see Fig. 4). The scaling relation between particle velocity amplitude and wave speed of Nesterenko solitary wave is


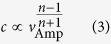


where 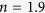 and thus 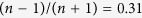. Fit of the simulation results with Eq. (3) is illustrated in Fig. 4, demonstrating that the investigated system satisfies Eq. (3) very well. In this strongly nonlinear system, the width of the wave (characteristic spatial length) *L*_n_, according to Nesterenko’s theory, can be given as


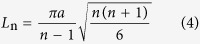


where *a* is the distance between adjacent particles (in our case *a* = *d*_0_). For buckyball system, Eq. (4) outputs *L*_n_≈3*a*, consistent with the simulation results. It is smaller than 5*a* in Hertzian chains with very small initial compression due to higher nonlinearity. Above analysis further confirms that this solitary wave obeys Nesterenko’s theory.

### Hexagonal close packing

**Figure 5:**
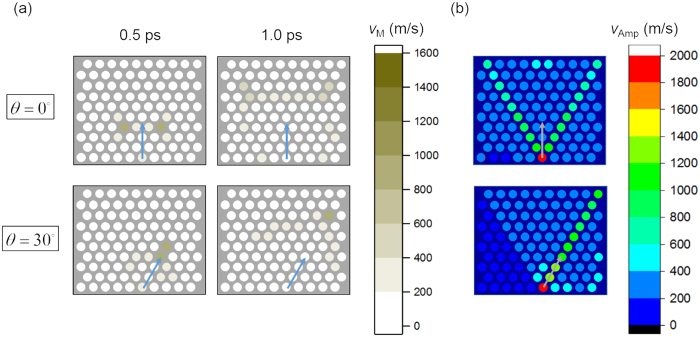
Stress wave distributions in *scp* system for different impacting angles. (**a**) Particle velocity magnitude distributions at given instants, i.e. 0.5 ps and 1.0 ps. (**b**) Particle velocity amplitude distributions.

Particle velocity magnitude distributions at given instants and particle velocity amplitude distributions of *hcp* configuration under two different impacting angles are presented in Fig. 5.

As can be seen, although stress wave propagation in *hcp* configuration tends to be directional, the amplitude decays as the wave travels and the input energy tends to be distributed. The reason is that in *hcp* system adjacent row and column will be affected by moving particles regardless of their velocity directions and thus energy is distributed to two or more surrounding particles. This pattern will continue spreading to more and more particles and energy or particle velocity amplitude is gradually decreasing. Therefore, *hcp* configuration of C_60_ molecules can serve as a nanoscale protective device.

**Figure 6:**
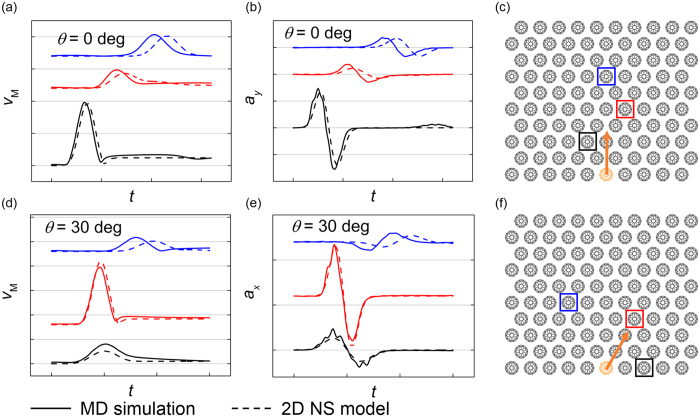
Comparison of particle velocity magnitude *v*_M_ and acceleration *a*_*x(y)*_ between MD simulations and numerical calculations in *hcp* granular configuration for different impacting angles. *v*_M_ and *a*_*x(y)*_ of the C_60_s in squares are extracted and the colors of the curves and the squares are corresponding. The vertical scale is 5 × 10^15^ m/s^2^ and the horizontal scale is 500 fs. (**a**), (**b**) and (**c**) *θ* = 0°. (**d**), (**e**) and (**f**) *θ* = 30°. The results of MD simulations and numerical calculations based on the 2D NS model are plotted in solid lines and dashed lines respectively.

The comparison between MD simulations and numerical calculations of *scp* system is shown in Fig. 6.

Although the agreement between the results of MD simulations and numerical calculations is generally good, it can be observed that there appears a little discrepancy between simulations and model predictions for the velocity and acceleration curves of some selected C_60_ molecules and it is not surprising due to the atoms vibrating about the equilibrium positions instead of being absolutely static after stress wave passes through. Therefore, the position or time of a C_60_ molecule when it interacts with other surrounding molecules is actually random. In addition, the parameters of NS model are obtained in 1D system where two degrees of freedom are eliminated, which may not be utterly suitable for 2D situation in this study. Due to error accumulation, the precision of model prediction decreases as the length of the force chain increases, and therefore, precisely predicting the velocity or acceleration of C_60_ molecule with a complex stress wave path can be fairly difficult. This also explains why the predictions on *scp* configurations are usually more accurate than *hcp* configuration on the condition that traveling distances of the stress waves are similar.

**Figure 7:**
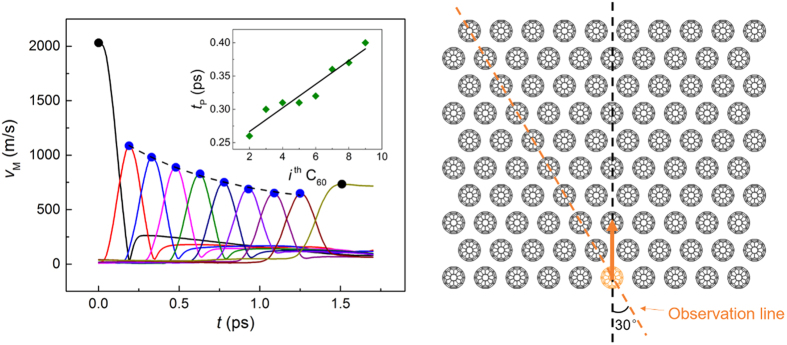
Stress wave attenuation along the observation line.

To further investigate the wave spreading behavior of nanoscale *hcp* configuration, a 30° observation line is defined (see the right panel of Fig. 7), along which stress wave propagation is studied. For a *θ* *=* 0° impact, the time histories of particle velocity magnitude of C_60_ molecules on the observation line is extracted and amplitudes of each molecule are marked, as is shown in the left panel of Fig. 7. Previous research on high dimensional-structured granular system tends to model the spreading of wave energy partition as exponentially decay^55^,^56^,^57^. In this study, we fit the particle velocity amplitude in the same manner and the fitting results are satisfactory (see the dashed line in Fig. 7). Moreover, the pulse duration *t*_p_ on each C_60_ molecule is subplotted in Fig. 7, showing that as the amplitude decreases, stress wave stays longer on a single C_60_ molecule.

## Concluding Remarks

At macroscale, orderly arranged granular materials have demonstrated its power to tune stress waves. At nanoscale, however, stress wave tuning and manipulations based on discrete granular configurations is a rather unexplored topic. In this work, we studied the 2D problem of nanoscale stress wave propagation as an extension of 1D propagation in buckyball system via MD simulation. We show that *scp* supports highly directional Nesterenko solitary waves along initially excited chain while *hcp* attenuates impact exponentially by continuously spreading wave energy to adjacent particles. We are able to describe the wave behaviors of both configurations in a good agreement with a 2D NS model. This work further validates the NS model as a feasible simplification of complicated van der Waals interaction in modeling the stress wave behaviors in nanoscale lattice of higher dimension and suggests the possibilities of 2D nanoscale system acting as energy harvesting, guiding or shock mitigating devices. More types of nanoscale granular configurations and their potential applications will be covered in our future work.

## Additional Information

**How to cite this article**: Xu, J. and Zheng, B. Stress Wave Propagation in Two-dimensional Buckyball Lattice. *Sci. Rep.* **6**, 37692; doi: 10.1038/srep37692 (2016).

**Publisher's note:** Springer Nature remains neutral with regard to jurisdictional claims in published maps and institutional affiliations.

## Supplementary Material

Supplementary Material
