# Supplementary material for: Stress Wave Propagation in Two-dimensional Buckyball Lattice
Source: Sci Rep. 2016 Nov 28;6:37692. doi: 10.1038/srep37692 (PMC5125272; doi:10.1038/srep37692)
Supplement: Supplementary Material [file srep37692-s1.doc]

# Supplementary Information

**Stress Wave Propagation in Two-dimensional Buckyball Lattice**

Jun Xu* and Bowen Zheng

Department of Automotive Engineering, School of Transportation Science and Engineering, Beihang University, Beijing, China, 100191

Advanced Vehicle Research Center, Beihang University, Beijing, China, 100191

*Corresponding author: Jun Xu (junxu@buaa.edu.cn)

# MD simulation descriptions

Carbon-carbon (C-C) interactions are modeled by Lennard-Jones (L-J) potential $U\left(r\right)=4\varepsilon\left[\left(\sigma/r\right)^{12}-\left(\sigma/r\right)^{6}\right]$, where *U* is the L-J potential for two atoms; *r* is the distance between atoms; *ε* and *σ* are two L-J parameters, representing the depth of the potential well and the finite distance for zero the inter-particle potential respectively, which are chosen as $\varepsilon=0.086\text{ kcal/mol}$ and $\sigma=3.400\mathop{\text{A}}\limits^{\text{o}}$.^1,2^ C-C bonds are modeled by Morse potential $E=D\left(1-\exp\left[-\alpha\left(r-r_{0}\right)\right]\right)^{2}$, where $D=114.46 \text{kcal}/\text{mol}$is the depth of the potential well; is the stiffness parameter; $r_{0}=1.416 \mathop{\text{A}}\limits^{\circ}$ is the equilibrium bond distance.^3,4^ Bond angles are modeled by a cosine/squared potential $E=K\left[\cos\left(\theta\right)-\cos\left(\theta_{0}\right)\right]^{2}\text{/2}$, where $K=134.37 \text{kcal/mol}$ is a prefactor and $\theta_{0}=120^{\circ}$ is the equilibrium value of the angle. In addition, a weak dihedral potential is applied to all bonded atoms. The time integration step is 1 fs. The system first runs for equilibrium in NVT ensemble (the canonical ensemble) at temperature $T=10\text{ K}$ for 3000 fs for relaxation and then run for 3000 fs in NVE ensemble for wave propagation. The out-of-plane degree of freedom of each C_60_ molecule is eliminated.

# 1D theoretical background

At macroscale, solitary wave is supported in 1D chains of homogeneous spherical granules and in the case of zero precompression, the interaction of adjacent granules has no linear term, leading to an interesting phenomenon of “sonic vacuum”, i.e. sound velocity in this granular media is zero.^5-7^ As a nanoscale counterpart, we studied 1D buckyball (C_60_) chain, where each molecule is at the equilibrium position, corresponding to the unprecompressed condition.^8^ We modeled the relatively complicated inter-molecular van der Waals interaction as a nonlinear spring and the equation of motion of *i*^th^ C_60_ molecule can be given as

|  | (1) |
| --- | --- |

where $\left(a\right)_{+}=\max\left(0,a\right)$; *u_i_* is the displacement from the initial position of *i*^th^ C_60_ molecule; *k* and *n* are stiffness parameter and nonlinearity index of N-S model ($k=5.6\times10^{10}$, $n=1.9$); *m* is the mass of a C_60_ molecule ($m=1.2\times10^{-24} \text{kg}$).

The relation between wave amplitude and wave speed is derived as

| $c_{\text{N}}=1.005\times A_{\text{N}}^{\frac{n-1}{2n}}$ | (2) |
| --- | --- |

where $n=1.9$ and therefore$\left(n-1\right)/2n=0.238$; $c_{\text{N}}$ is normalized wave speed, normalized by $c_{0}=5181 \text{m}/\text{s}$; $A_{\text{N}}$ is normalized amplitude, normalized by $A_{0}=4.29 \text{nN}$。

**Table S1. Force amplitudes of excited solitary waves for various impacting angles.**

| Impacting angle* θ* (deg) | 0 | 15 | 30 | 45 | 60 | 75 |
| --- | --- | --- | --- | --- | --- | --- |
| Force amplitude *A* (nN) | 2.81 | 6.01 | 9.32 | 12.5 | 14.5 | 15.5 |

For fully developed solitary waves, force amplitude remains almost constant as the wave travels. Therefore, the force amplitude in Table S1 is the average value of those recorded on 3^rd^ to 8^th^ C_60_s.

| 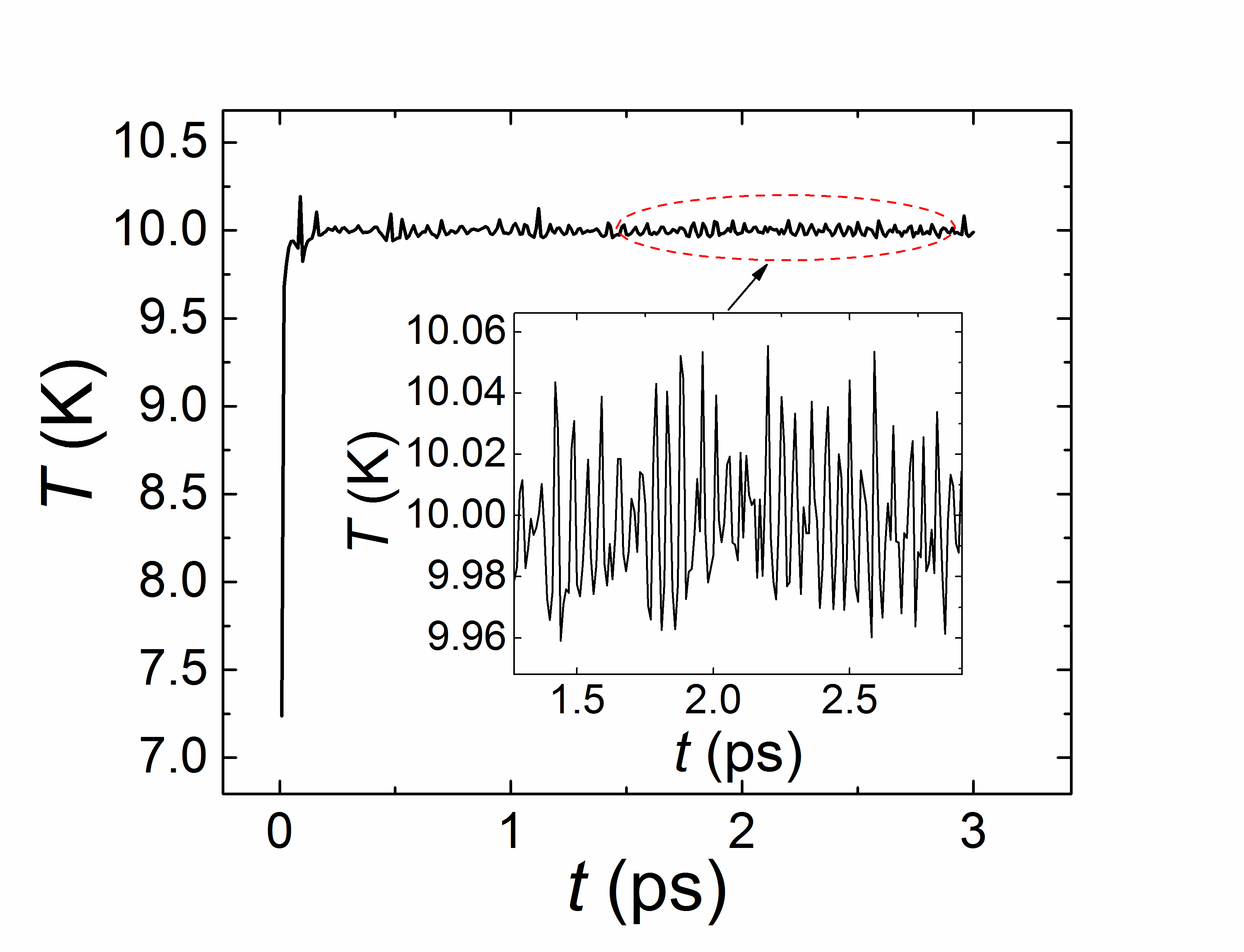 |
| --- |
| **Figure S1. Running for equilibrium in NVT.** |

| 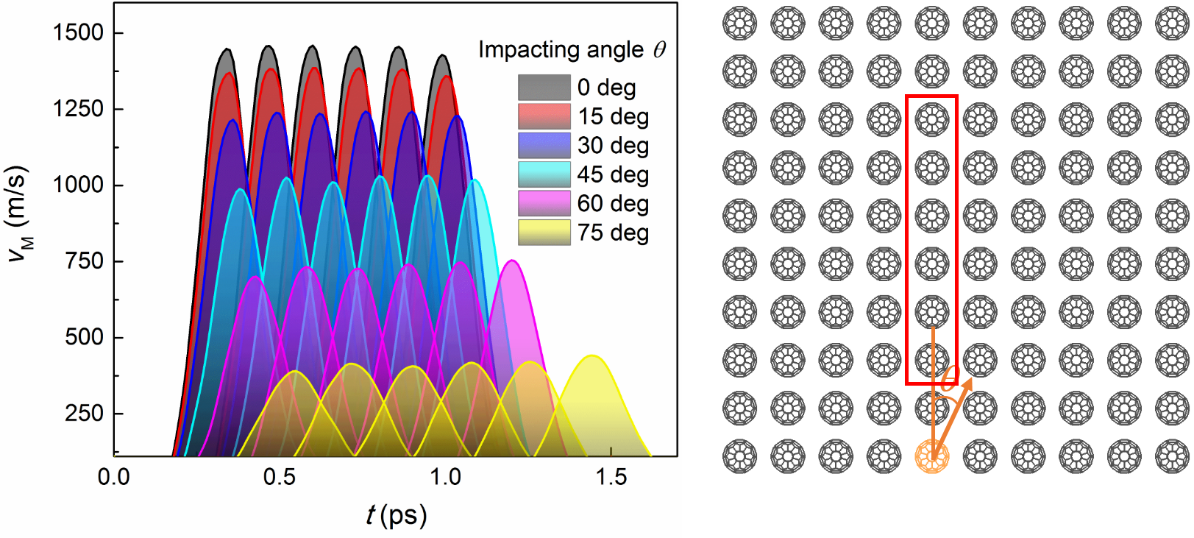 |
| --- |
| **Figure S2. Wave shape for *scp* configuration under various impacting angles from 0 deg to 75 deg.** Particle velocity magnitude histories of 6 C_60_ molecules highlighted by red square are recorded. As is clearly shown, wave speed is amplitude-dependent, which is a typical feature of nonlinear waves. |

| 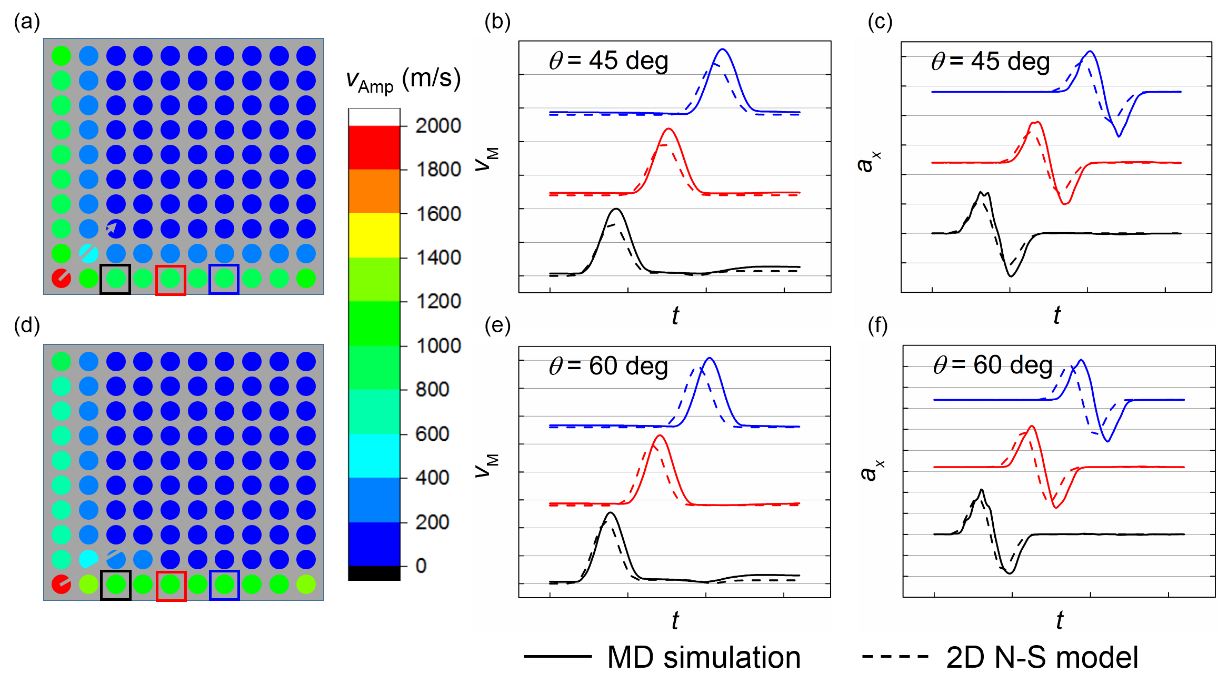 |
| --- |
| **Figure S3. Model validation for a different choice of impactor position for *scp* configuration.** The results of MD simulations and numerical calculations based on N-S model are presented in solid and dashed lines respectively. $v_{\text{M}}$ and $a_{x\left(y\right)}$ of the C_60_s in squares are extracted and the colors of the curves and the squares are corresponding. The vertical scale is $5\times10^{15} \text{m}/\text{s}^{2}$ and the horizontal scale is 500 fs. (a), (b) and (c) $\theta=45^{\circ}$. (d), (e) and (f) $\theta=60^{\circ}$. The results of MD simulations and numerical calculations based on the 2D N-S model are plotted in solid lines and dashed lines respectively. |

**References**

1 Jones, J. E. On the Determination of Molecular Fields. II. From the Equation of State of a Gas. *Proc. R. Soc. London Ser. A* **106**, 463-477, doi:10.1098/rspa.1924.0082 (1924).

2 Hummer, G., Rasaiah, J. C. & Noworyta, J. P. Water conduction through the hydrophobic channel of a carbon nanotube. *Nature* **414**, 188-190 (2001).

3 Morse, P. M. Diatomic Molecules According to the Wave Mechanics. II. Vibrational Levels. *Phys. Rev.* **34**, 57-64 (1929).

4 Lide, D. R. *CRC handbook of chemistry and physics*. Vol. 85 (CRC press, 2004).

5 Nesterenko, V. F. Propagation of nonlinear compression pulses in granular media. *J. Appl. Mech. Tech. Phys.* **24**, 733-743, doi:10.1007/bf00905892 (1983).

6 Lazaridi, A. & Nesterenko, V. Observation of a new type of solitary waves in a one-dimensional granular medium. *J. Appl. Mech. Tech. Phys.* **26**, 405-408 (1985).

7 Coste, C., Falcon, E. & Fauve, S. Solitary waves in a chain of beads under Hertz contact. *Phys. Rev. E* **56**, 6104-6117 (1997).

8 Xu, J., Zheng, B. & Liu, Y. Solitary Wave in One-dimensional Buckyball System at Nanoscale. *Sci. Rep.* **6**, 21052, doi:10.1038/srep21052 (2016).
